# PSOLA: A Heuristic Land-Use Allocation Model Using Patch-Level Operations and Knowledge-Informed Rules

**DOI:** 10.1371/journal.pone.0157728

**Published:** 2016-06-20

**Authors:** Yaolin Liu, Jinjin Peng, Limin Jiao, Yanfang Liu

**Affiliations:** 1 School of Resource and Environment Sciences, Wuhan University, 129 Luoyu Road, Wuhan 430079, China; 2 Key Laboratory of Geographic Information Systems, Ministry Education, 129 Luoyu Road, Wuhan 430079, China; 3 Key Laboratory of Digital Mapping and Land Information Application Engineering, National Administration of Surveying, Mapping and Geoinformation, 129 Luoyu Road, Wuhan 430079, China; 4 Collaborative Innovation Center for Geospatial Information Technology, Wuhan University, 129 Luoyu Road, Wuhan 430079, China; Peking UIniversity, CHINA

## Abstract

Optimizing land-use allocation is important to regional sustainable development, as it promotes the social equality of public services, increases the economic benefits of land-use activities, and reduces the ecological risk of land-use planning. Most land-use optimization models allocate land-use using cell-level operations that fragment land-use patches. These models do not cooperate well with land-use planning knowledge, leading to irrational land-use patterns. This study focuses on building a heuristic land-use allocation model (PSOLA) using particle swarm optimization. The model allocates land-use with patch-level operations to avoid fragmentation. The patch-level operations include a patch-edge operator, a patch-size operator, and a patch-compactness operator that constrain the size and shape of land-use patches. The model is also integrated with knowledge-informed rules to provide auxiliary knowledge of land-use planning during optimization. The knowledge-informed rules consist of suitability, accessibility, land use policy, and stakeholders’ preference. To validate the PSOLA model, a case study was performed in Gaoqiao Town in Zhejiang Province, China. The results demonstrate that the PSOLA model outperforms a basic PSO (Particle Swarm Optimization) in the terms of the social, economic, ecological, and overall benefits by 3.60%, 7.10%, 1.53% and 4.06%, respectively, which confirms the effectiveness of our improvements. Furthermore, the model has an open architecture, enabling its extension as a generic tool to support decision making in land-use planning.

## 1. Introduction

Land-use allocation is a process of allocating different activities or uses to specific units of area within a geospatial context, to maximize a spectrum of social, economic, and ecological benefits [1]. It is a complicated resource allocation problem involving large volumes of data, complex spatial operations, and multi-objective trade-offs. There are tons of land use simulation models in literatures such as cellar automaton (CA) [2], Land Use Scanner [3], CLUE-S [4], Land Transformation Model (LTM) [5], Classification And Regression Trees (CART) and Multivariate Adaptive Regression Splines (MARS) [6]. However, these simulation models are aim to predict future land use, not to optimize land use spatial configuration. These models are limited to optimize land use for only generate few land use scenarios. Optimization methods, such as exact methods and heuristic methods, can generate much more land use schemes to search for a better solution. In this paper, we focus on land use models with optimization methods.

Exact methods, such as linear programing (LP) [7–11] and integer programing (IP) [12–15], have been utilized to solve land-use allocation problems. However, land-use allocation is always a non-linear, multi-peak, geospatial-related problem. In most cases, it is impossible to generate optimal solutions using exact methods over a large area with non-linear objectives. Such complicated non-linear multi-objective optimization problems, as a form of non-deterministic polynomial (NP) hard problem, require heuristic methods for executing the optimization processes [1].

Heuristic methods are capable of generating near-optimal solutions with an acceptable time cost. They have no limitations to the form of the objectives and constraints. Many land-use allocation models have been developed using heuristic methods: Aerts [16] applied simulated annealing (SA) to the multi-objective site selection problem; Cao and Batty [17] utilized a genetic algorithm (GA) to generate several optimized solutions in urban land-use allocation; Liu [18] introduced ant colony optimization (ACO) for zoning protected ecological areas; and Hu [19] developed a particle swarm optimization (PSO) model for the optimal allocation of earthquake emergency shelters.

Most land use optimization models with heuristic methods, however, allocate land use using cell-level operations. These models divide land-use patches into isolated grids to allocate land use cell by cell. When the models operate at a fine resolution such as 25m * 25m, 50m * 50m, fragmentation happens. In practice, local authorities manage land use at the patch level; therefore, there is a gap between theory and practice in the level of operations. Masoomi [20] developed a model to optimize urban land-use at the patch level using polygon-representation. But the model was limited to combining, splitting, or otherwise modifying the shapes of land use patches, whereas these modifications are potentially desirable in practice [21]. Liu [22] used cell-level operations to modify land use patches with grid-representation. The cell-level operations with a fine resolution overlooked patch-level constraints, breaking down land-use adjacency and connectivity. Fragmented land-use was observed in some local areas. It is a challenge to reconcile the desire of modifying land use patches and the demands of keeping land use adjacency and connectivity.

Another deficiency in these land use optimization models is the absence of land-use planning knowledge. The models are stochastic without possessing an understanding of the underlying mechanisms of land-use change [18]. Land-use transitions are heavily reliant on transition probabilities, which inevitably misdirect land-use conversions in certain local areas. Land-use planning knowledge such as conversion costs, suitability, and land use policies should be integrated to help the optimization models to do land use transitions. Liu [23] proposed a model integrated with social-economic driving forces to transform land use, but the model only utilized the driving forces to adjust transition probabilities, while the transitions were still random. Liu [24] coordinated land-use competitions using game theory to direct land-use transitions, but the coordination of incompatible land uses was a form of post-processing that was loosely coupled in the model, which is hard to operate in practice. Other modellers draw attention to the design of various encoding schemas and spatial operators, without realizing the importance of land-use planning knowledge [17, 25–28].

This study focuses on building a heuristic land-use allocation model using particle swarm optimization (PSO). To overcome the aforementioned shortcomings, the model is integrated with patch-level operations and knowledge-informed rules. The patch-level operations rebuild land-use patches from land-use grids, making it possible to implement patch-level constraints. The patch-level operations include a patch-edge operator, a patch-size operator, and a patch-compactness operator that constrain the size and shape of land-use patches. With patch-level operations, the model is capable of optimizing at the patch level without losing the ability of land-block modification.

Knowledge-informed rules provide priori knowledges to help optimization models generate better land use scheme with less time consumption. Empirical evidence indicates that spatial allocation problems can be solved faster and more easily using knowledge-informed rules [29]. Knowledge-informed spatial optimization is a promising approach to improving the efficiency and effectiveness of spatial allocation solutions [30]. In this study, knowledge-informed rules are infused to provide the domain knowledge of land-use planning during optimization. We consulted departments of land management and experts in relevant fields to acquire a knowledge base of land-use planning. A set of knowledge-informed rules was constructed on the aspects of suitability, accessibility, land use policy, and stakeholders’ preferences.

In this article, a land-use allocation model (PSOLA) with particle swam optimization is proposed to assist with decision-making in land-use planning. The rest of the article is organized as follows. Section 2 formulates the land-use allocation problem. The techniques to build the model, especially the patch-level operations and the knowledge-informed rules, are described in Section 3. In Section 4, we applied the model in a study area, and the results and discussion are presented in the following section. Finally, the conclusions and future work are presented in Section 6.

## 2. Land-Use Allocation Problem Formulation

Land-use allocation is a type of resource allocation problems (RAP) [31]. The resource is the land, which is subdivided into a finite number of units within a region. The purpose is to allocate various land use activities to suitable locations to maximize a spectrum of social, economic, and ecological objectives. A land-use allocation problem can be defined as [32, 33]:

M=(X,S,Ω,F) (1)

where *X* is the decision variable, which stands for the land-use type of each spatial unit. *S* is the solution space representing all feasible land-use alternatives. When allocating *K* types of land-use to *N* spatial units, *S* increases to *N^K*.*Ω* are the spatial and non-spatial constraints in the allocation, and *F* is a spectrum of objectives. The goal of optimizing the land-use allocation is choosing some solutions *s*_*opt*_ that satisfy the condition:

$$F(s_{opt})\geq F(s),\forall s\in S$$ (2)

In this study, the land-use allocation problem is described as allocating *K* land-use types to a grid space with *R* rows and *C* columns, to maximize the land-use benefits *F*. *F* is a vector of objectives *f* that evaluates the social, economic, and ecological benefits of the candidate solutions. The land use type of each cell is represented by a binary variable *x*_*ijk*_. When cell (*i*, *j*) is allocated with land-use *k*, *x*_*ijk*_ = 1; otherwise *x*_*ijk*_ = 0 (Eq 4). The formulation of the land-use allocation problem is:

$$Maximize:F(x)=(f_{1}(x),f_{2}(x),\cdot \cdot \cdot ,f_{N}(x))$$ (3)

$$Subject\;to:x_{ijk}\in \left\{0,1\right\},\forall i\in R,\forall j\in C,\forall k\in K$$ (4)

$$\overset{K}{\underset{k=1}{\sum }}x_{ijk}=1$$ (5)

$$L_{k}\leq \overset{R}{\underset{i=1}{\sum }}\overset{C}{\underset{j=1}{\sum }}x_{ijk}=Q_{k}\leq U_{k}$$ (6)

$$x_{{}_{ijk}}\subset \Omega_{k}$$ (7)

Eq 5 regulates that a land unit can be allocated with only one land-use type. Eq 6 defines the lower bound *L* and the upper bound *U* of each land-use type. The quantity of each land-use is prescribed by the local bureau of land-use planning, which balances the land-use supply and demand for future development. Eq 7 states that the land use allocation should comply with other spatial and non-spatial constraints such as soil conditions, transportation requirements, and land use policy.

The land use benefits *F* involve a set of sustainability objectives related to social equality, economic development, and ecological protection [27]. To constitute a specific understanding of sustainable development, some metrics are proposed to quantify these objectives:

### Maximization of social benefit

A social sustainable system should emphasize the well-being of people and their communities to promote social equality in living, health and education [27]. Compact land use leads to the effective utilization of land resources, increasing the accessibility and convenience to public services. Conversely, extremely compact land-use is undesirable, as it causes a reduction in dwelling size, health risk from overcrowding, and higher crime rates associated with high-density living [34]. Determining the compactness that is sustainable is highly subjective, involving combinations of people's preferences on population density and commuting [35]. China is promoting the building of a new socialist countryside, aiming to improve the living environment in rural areas and accelerating development of rural public services. Scattered rural settlements are consolidated for compact land use, so the compactness of rural settlements is used to quantify the social benefit. The basic neighbour method is chosen to evaluate the compactness for simplicity (Eq 8).

$$f_{social}=\overset{}{\underset{(i,j)\in \Omega }{\sum }}x_{ijk}/N_{\Omega }$$ (8)

where Ω is the neighbours of the focused cell, usually a 3*3 Moore neighbourhood.

### Maximization of economic benefit

GDP is a widely used metric to evaluate economic benefit. However, it is hard to estimate the profit of each land use, as it varies with location and time. The urban area is a major contribution to the economic growth, whereas unlimited urban expansion would cause many side effects, such as a grain production decrease and environmental pollution. Generally, the local government sets an urban growth boundary to control urban expansion and ensure the demands of economic development. So, the economic benefit is evaluated by Eq 9.

$$f_{economic}=e^{-\frac{{\left(x-\mu \right)}^{2}}{2\sigma^{2}}}$$ (9)

Eq 9 is a normal distribution with the expectation of *u*, where *u* is the permissible maximum urban area. *σ*is a stretch factor controlling the extent of the normal distribution.

### Maximization of ecological benefit

Forests provide enormous ecological benefits by reducing air pollution, slowing storm water runoff, helping conserve energy, and providing wildlife habitats. The forest core area is the interior area of a homogenous mature forest patch, which is very important because many species primarily inhabit or reach their highest abundance in the forest core area. The core area index (CAI) of a forest is utilized to quantify the ecological benefit, which equals the percentage of the core area (Eq 10),

$$f_{eco\text{log}ical}=N_{core}/N_{forest}$$ (10)

where *N*_*core*_ is the area of the forest core area, and *N*_*forest*_ is the total area of the forest.

Based on the previous metrics, the fitness function (Eq 11) is constructed using a weighted sum method.

$$F=w_{1}•f_{social}+w_{2}•f_{economic}+w_{3}•f_{ecological}$$ (11)

where *w*_1_ + *w*_2_ + *w*_3_ = 1.

## 3. Specification of the PSOLA Model

**Fig 1:**
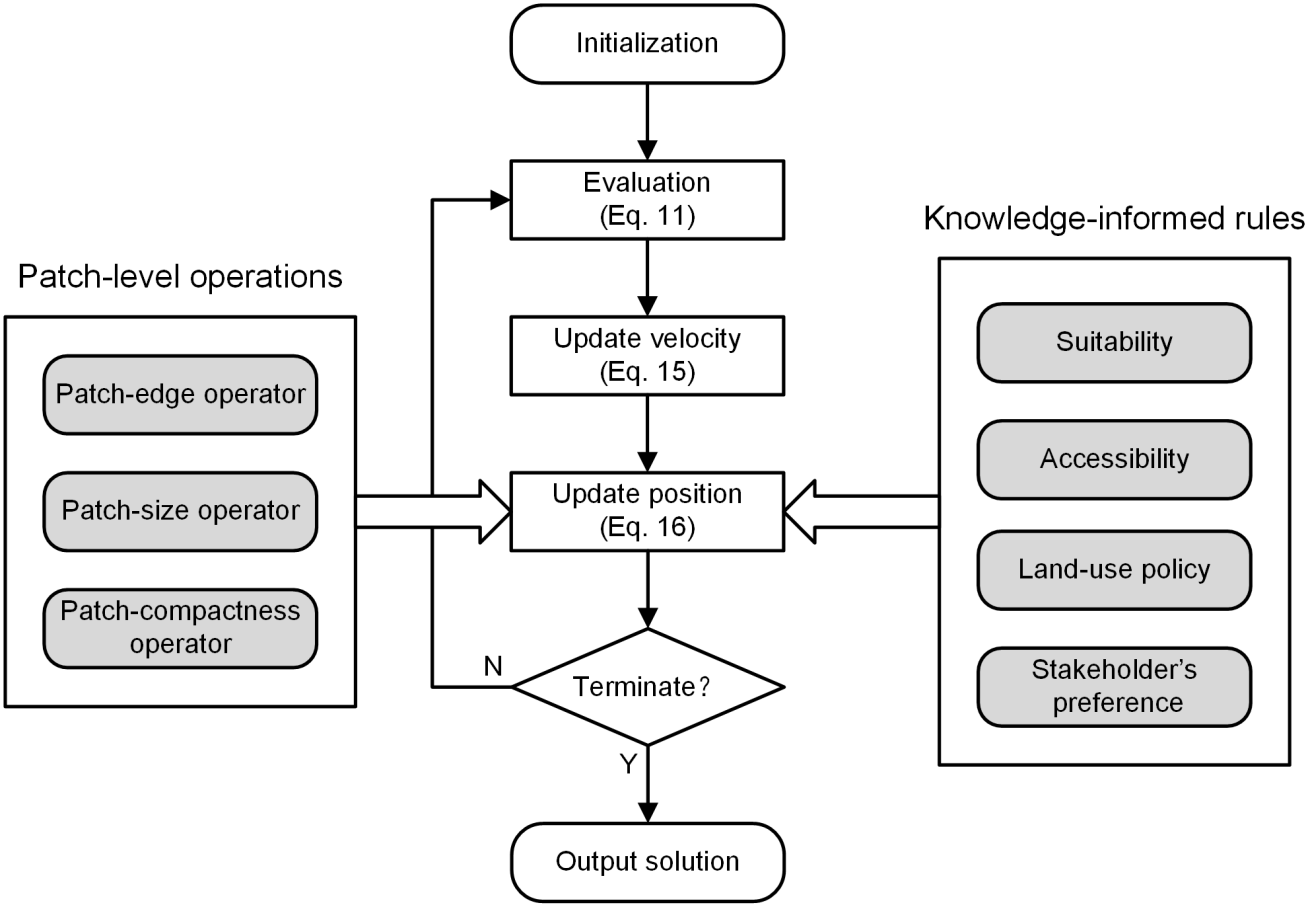
Optimizing procedure of the PSOLA model.

In this section, we will provide the specifications to build the PSOLA model. The procedure for optimizing land-use allocation using patch-level operations and knowledge-informed rules is illustrated in Fig 1. First, a set of particles is initialized on the basis of the actual land-use map. Each particle denotes a candidate land-use spatial pattern. Then, the particles are evaluated by a fitness function composed of allocating objectives. Every particle updates its velocity and position through a learning procedure from its own experience and those of its companions. The learning procedure is iterated until some terminal conditions such as maximum generations or time consumption are fulfilled. To improve the ability of searching for optimal land-use allocation schemes, the model is integrated with patch-level operations and knowledge-informed rules. The patch-level operations equip the model with patch-level optimization and the knowledge-informed rules direct rational land-use transitions. More details will be provided in Section 3.2 and Section 3.3.

### 3.1 A new discrete PSO for land-use allocation

Particle swarm optimization is inspired by the scattering and regrouping behaviour in bird flocking or fish schooling [31]. It is a population and evolution based optimization technique using a heuristic search [23]. In PSO, a candidate solution for a specific problem is called a particle. Each particle moves through the multi-dimensional problem space with a velocity that is dynamically adjusted by its own experience and those of its companions [36]. Finally, particles cooperate on exploring the problem space to find near-optimal solutions.

In a *D*-dimensional search space, the population is *I*. For the *i*-th particle in the *d*-th dimension, its position and velocity are *x* and *v*, respectively. The best position found by the particle is the personal best *p*_*i*_, and the best position of the swarm is the global best *p*_*g*_. At time step *t*, the particle updates its velocity and position as

$$v(t+1)=wv(t)+c_{1}r_{1}(p_{i}-x(t))+c_{2}r_{2}(p_{g}-x(t))$$ (12)

x(t+1)=x(t)+v(t+1) (13)

where *w* is the inertia weight, which is employed to maintain the current speed; *c*_*1*_ is the cognitive learning coefficient, which controls the tendency of approaching *p*_*i*_; *c*_*2*_ is the social learning coefficient, which control the tendency of approaching *p*_*g*_; *and r*_*1*_ and *r*_*2*_ are random numbers in [0, 1]. The particle is then evaluated by a fitness function. The PSO algorithm iterates the process until reaching terminal conditions such as maximum generations or time consumption.

**Fig 2:**
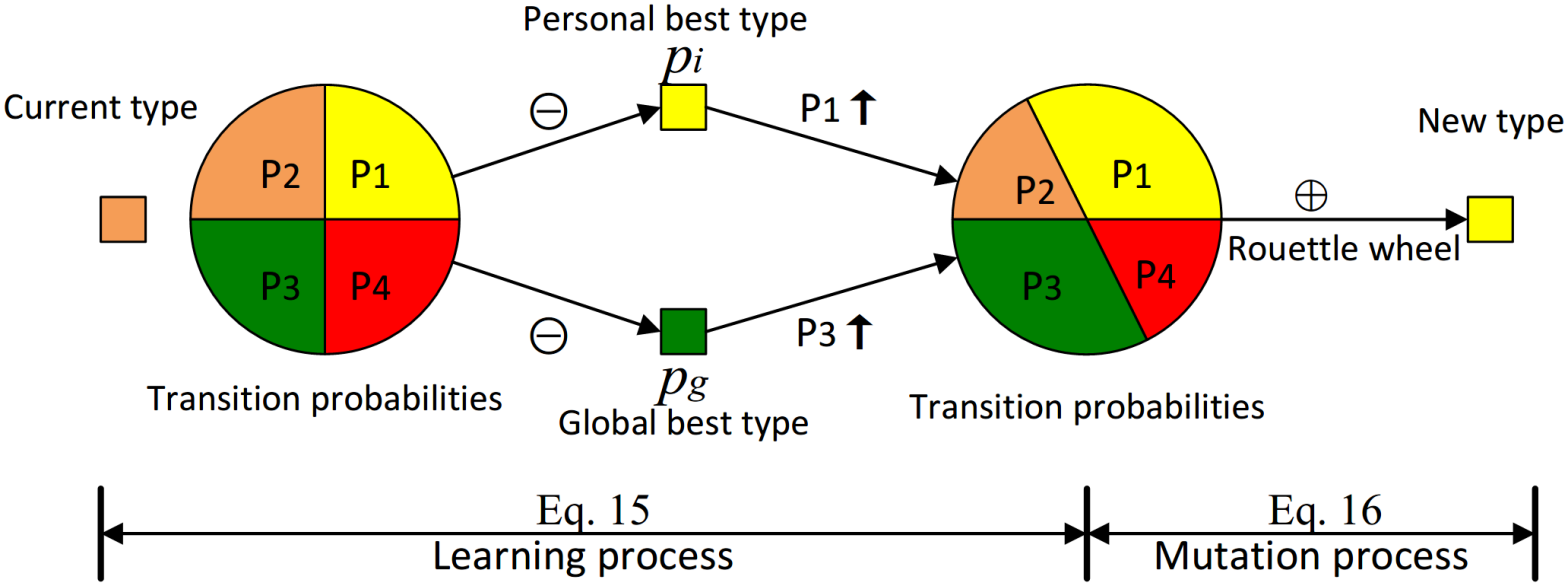
Process of a spatial unit converting its current land-use type to a new type.

The aforementioned algorithm is the basic PSO meant to solve problems in a continuous solution space. However, the land-use allocation is a combinatorial optimization problem with a discrete solution space [37]. To build a discrete PSO, the velocity *v* is modified to a *K*-dimensional (*K* is the number of all land-use types) vector *v*, which represents transition probabilities (Eq 14). The transition process is illustrated in Fig 2. A land unit updates its transition probabilities through a learning process of the personal best type *p*_*i*_ and the global best type *p*_*g*_ (Eq 15). Then, the land unit converts to a new land-use type through a mutation process with a roulette wheel based on its transition probabilities (Eq 16).

$$\vec{v}(t)=\left[P_{1},P_{2},\cdot \cdot \cdot ,P_{K}\right]$$ (14)

$$\vec{v}(t+1)=w\vec{v}(t)+c_{1}r_{1}(p_{i}⊖x(t))+c_{2}r_{2}(p_{g}⊖x(t))$$ (15)

$$x(t+1)=x(t)⊕\vec{v}(t+1)$$ (16)

where ⊝ is a learning operator, which acts to increase the probability of transforming to the personal best type *p*_*i*_ and the global best type *p*_*g*_, and ⊕ is a mutation operator, which mutates to a new land-use type by a roulette wheel.

### 3.2 Patch-level operations

**Fig 3:**
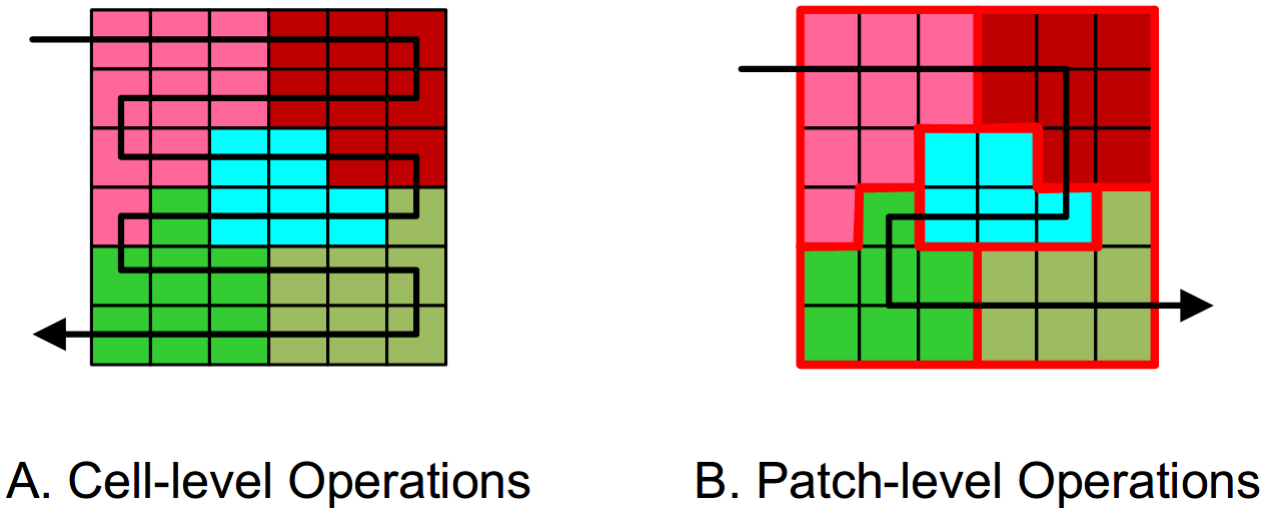
Cell-level operations (A) versus patch-level operations (B).

In previous works [24, 38, 39] on a heuristic model for land-use allocation using raster datasets, they allocated land-use with cell-level operations (Fig 3A). Land parcels are divided into isolated cells to be allocated cell by cell. This method of allocation has two main disadvantages. First, algorithm suffers from low efficiency when the model is employed in a large region, as the computational burden increases exponentially as the area grows. Many researchers have found that a model takes hours to generate satisfactory solutions [25, 27]. Second, the model breaks down land-use adjacency and connectivity, causing fragmentation and a low-intensity land-use pattern.

**Fig 4:**
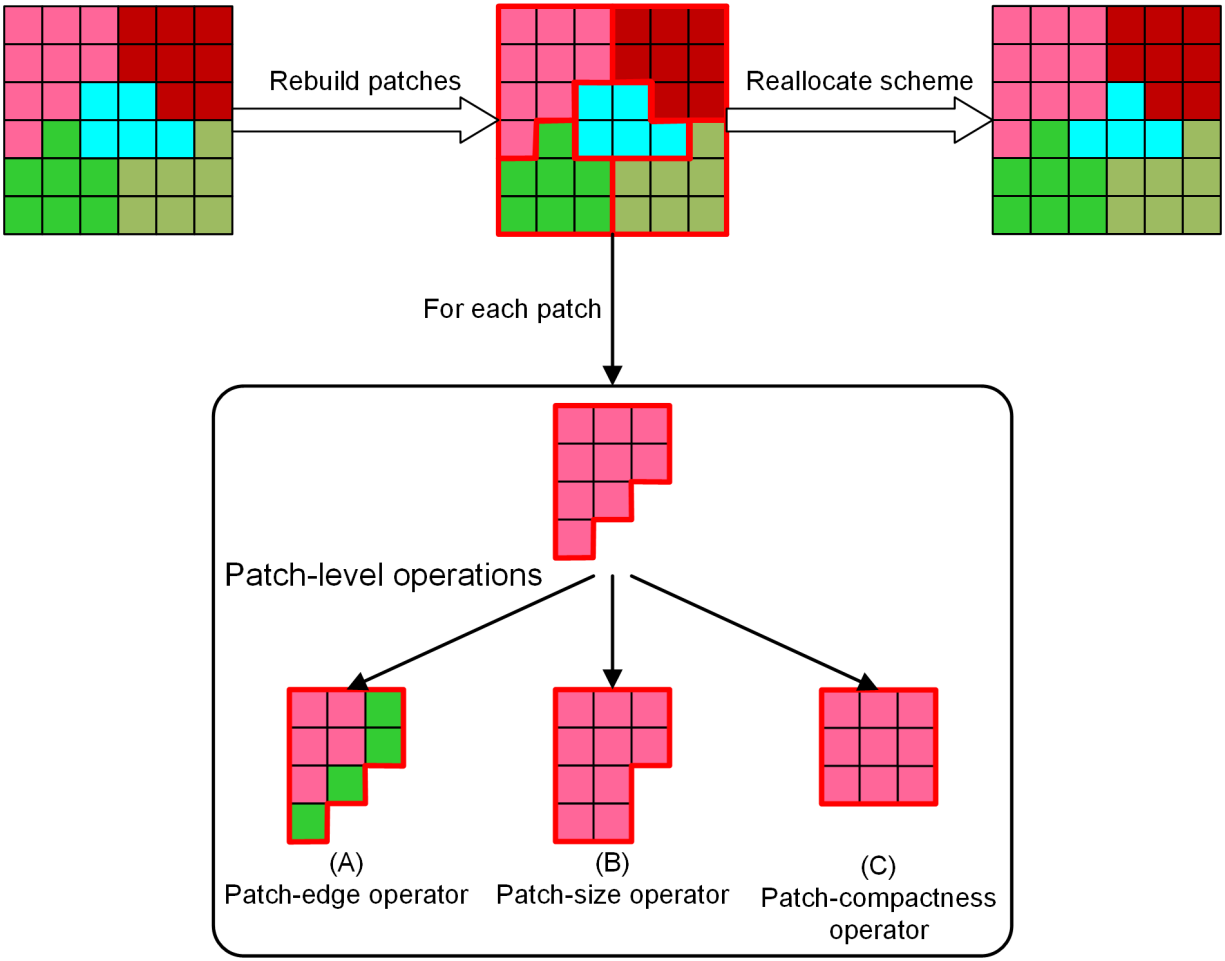
Procedure of allocating land use with patch-level operations.

Considering the disadvantages of cell-level operations, the PSOLA model allocates land use using patch-level operations (Fig 3B). The procedure of allocating land use with patch-level operations is illustrated in Fig 4. To support patch-level operations, the model first rebuilds land-use patches by reclassifying land grids. Then, an upper level interface is provided for modelers to design spatial operators. In this research, we designed three patch-level operators: a patch-edge operator, a patch-size operator, and a patch-compactness operator. The operators constrain the size and shape of land-use patches during optimization. Further details on these operators will be provided in the following paragraph. Finally, a land use scheme is reallocated until all patches are optimized by the patch-level operators.

#### Patch-edge operator (Fig 4A)

In landscape ecology, it is a consensus that many ecosystem processes respond differently to the interior and exterior of a forest parcel [40]. This fact inspired us to suppose that land use activities also react differently to the core and edge of a land-use patch. The core area is more stable due to its homogeneous surroundings, whereas the edge area is volatile because of the edge effects at the boundary. The patch-edge operator was designed to vary the reactions in the core and edge area. The operator splits a land use patch into two distinct areas: a core area and an edge area. The core area is the inner part of a land-use patch and the edge area is the boundary part. The operator only permits land-use transitions in the edge area, while keeping the core area unchanged. During optimization, a land use patch would expand or shrink from the edge area.

#### Patch-size operator (Fig 4B)

Patch size strongly governs the effectiveness of land use activities. Large arable land patches are propitious for mechanized farming, and large orchards are convenient to be managed uniformly. Patch size can also affect species habitats, resource availability, competition, and recolonization. Many species are sensitive to the size of the habitat because they need an area large enough to hunt for food. The patch-size operator constrains the size of a land use patch, to guarantee that an area is large enough to support its land use activities.

#### Patch-compactness operator (Fig 4C)

Compact land use increases the accessibility and convenience of public services, leading to an intensive land-use pattern. The patch-compactness operator optimizes the shape of a land use patch to increase the compactness of the patch. A shape index (Eq 17) is used to evaluate the compactness of a patch.

$$SHAPE=p_{i}/\text{min}\;p_{i}$$ (17)

where *p*_*i*_ is the perimeter of land use patch *i*, and min *p*_*i*_ is the minimum perimeter of this patch.

### 3.3 Knowledge-informed rules

Many heuristic land-use allocation models such as the genetic algorithm (GA) [1, 17, 28, 41], ant colony optimization (ACO) [18], artificial immune systems (AIS) [33, 42] and particle swarm optimization (PSO) [19, 20, 22], are global optimization models that pursue overall optimal solutions to satisfy a host of global objectives. Those global optimization models are limited in their understanding of the underlying mechanisms of land-use change [38], so the domain knowledge of land-use planning should be integrated into the allocation models to guide land-use transitions.

The PSOLA model introduces knowledge-informed rules to provide land-use planning knowledge. According to Eq 16, the mutation process is driven only by transition probabilities. It is a stochastic process leading to irrational land-use transitions. Hence, it is necessary to regulate these random land-use transitions using land-use planning knowledge to ensure a rational land use pattern. Knowledge-informed rules are the auxiliary knowledge of the nature and structure of spatial configurations. Knowledge-informed spatial optimization is a promising approach to improving the efficiency and effectiveness of spatial allocation solutions. The PSOLA model integrates the knowledge-informed rules *T* to guide the land-use transitions during optimization. The mutation process is modified to consider the coupled effects of the transition probabilities and knowledge-informed rules (Eq 18).

$$x(t+1)=x(t)⊕\vec{v}(t+1)⊕T$$ (18)

The definition of knowledge-informed rules is a challenging task because of the many spatial variables and parameters involved [43]. There are many ways to define knowledge-informed rules such as artificial neural network (ANN), classification and regression trees (CART), multivariate adaptive regression splines (MARS) [6], multi-criteria evaluation (MCE) [44], ant colony optimization (ACO) [45], and artificial immune system (AIS) [46]. In the PSOLA model, we used a simple and direct method to acquire knowledge-informed rules. A questionnaire survey was distributed to the Bureau of Land and Resources, the Bureau of Land Use Planning, and experienced experts in agriculture, forestry, and ecology, and we analyzed their suggestions to produce a set of knowledge-informed rules. The knowledge-informed rules consist of suitability, accessibility, land use policy, and stakeholders’ preferences, showed in Table 1:

**Table 1:** Knowledge-informed rules for land-use allocation.

Category	Rule	If	Then
Suitability	Suitability	Target land-use type = arable land, orchard, forest, or grass AND Suitability = highly suitable or suitable	OK for target land-use
Accessibility	Farming radius	Target land-use type = arable land AND Distance to residential area ≤ 1000m	OK for arable land use
	Transportation	Target land-use type = orchard AND Distance to roads ≤ 500m	OK for orchard land-use
Land-use policy	Grain for Green	Current land-use type = arable land AND Slope ≥ 25 degrees	Convert to forest land use
	Max area	Area of target land-use type < Q_*k*_ ^a^	OK for target land-use
	Undeveloped area	Current land-use type = grass, developed urban, mining area, roads, water, or barren land	Keep current land-use status
	Soil and water conservation	Current land-use type = forest AND Distance to river ≤ 50m	Protected for forest land-use
Stakeholders’ preferences	Rural settlement consolidation	Current land-use type = rural settlement AND Patch size ≤ 10000 m^2^	Move to the nearest centralized residential area
	Density based design constraint ^b^	Neighborhood with target land-use type ≥ 4	OK for target land use

**^a^** Q_*k*_ is predefined by local general land-use planning.

**^b^**Density-based design constraint (DBDC) is a spatial operator developed by Ligmann-Zielinska [47, 48] that facilitates compact neighborhood development.

According to the aforementioned techniques, we implemented the PSOLA model in C++. The model uses the geospatial data abstraction library (GDAL) to access GIS data. The model is also paralleled with the message passing interface (MPI) for efficiency. We open-sourced the model on GitHub (http://jingsam.github.io/PSOLA/) so that anyone who is interested in land-use allocation using a PSO algorithm could use, modify or share it for free.

## 4. Case Study

### 4.1 Study area

**Fig 5:**
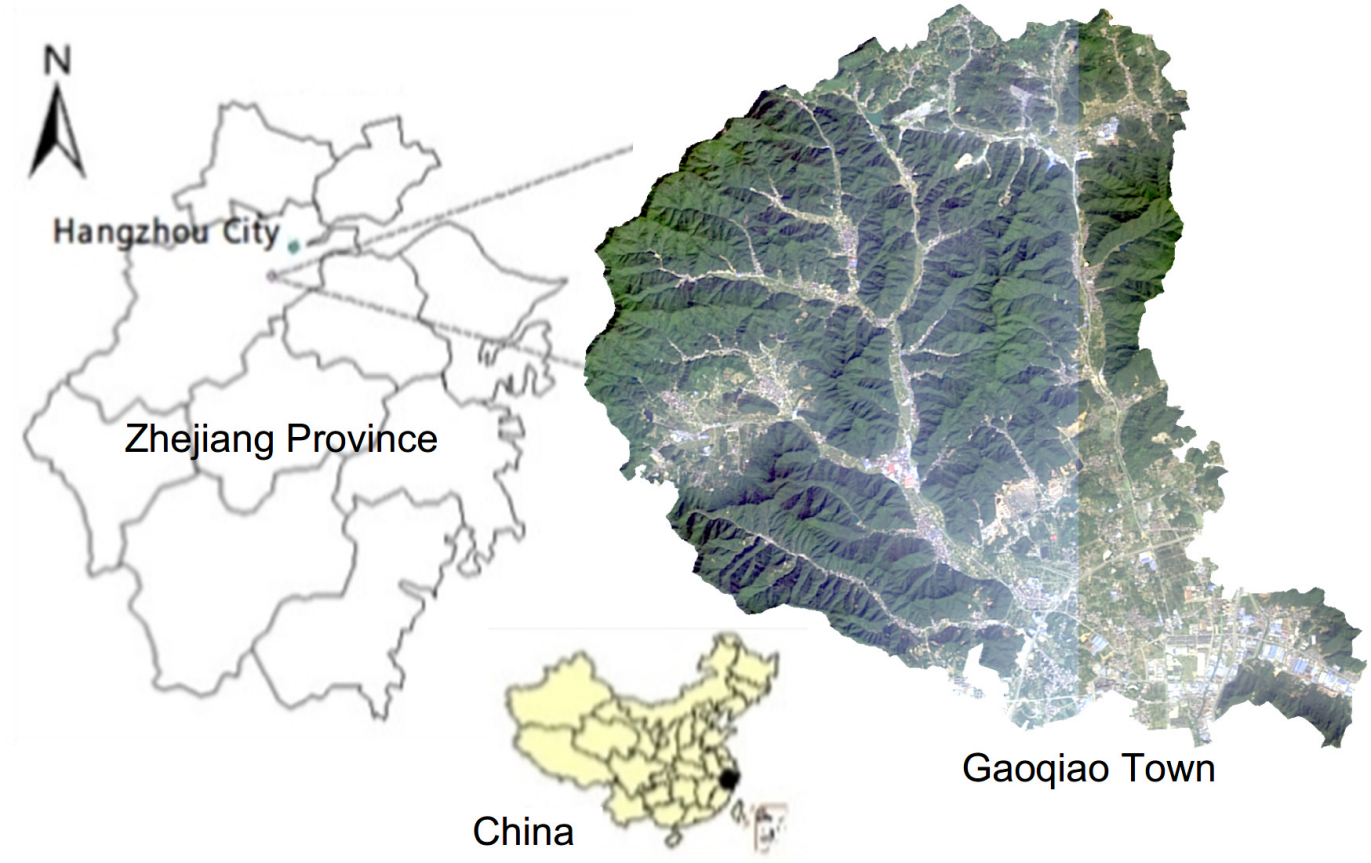
Study area.

Gaoqiao, an eastern coastal town in China, was taken as the study area. Gaoqiao is located 20 km to the southwest of Hangzhou City in Zhejiang Province (Fig 5). The landscape of this region is mainly made up of plains and hills. The area has a monsoon climate of the subtropical temperature zone, enjoying a temperate climate with plenty of rainfall and sunshine. Gaoqiao has 16 administrative villages, with a total area of 104.03 square kilometres and a population of 58600.

Gaoqiao boasts a rapidly developing economy and has become one of the most developed towns in China. However, its blooming economic development activities also cause side effects. A large amount of cultivated land has been arbitrarily converted to construction land to fulfil the demands of economic development. This activity has caused some undesirable phenomena, such as land waste, soil pollution, and ecological damage. For the purpose of sustainable development, there is an urgent need to optimize the land-use allocation to balance the benefits of social equality, economic development, and ecological protection.

### 4.2 Data processing

The data related to the land-use allocation are presented in Table 2. The land-use map produced by the Second National Land Survey was used as the base map. The period of land-use planning is 2009–2020. Data are fused to the year 2009. A grey model GM(1,1) was used to predict the population in 2020 according to the socioeconomic data from 1996–2010. All spatial data were rasterized or resampled to a resolution of 25 m. Each map was converted to a 515*545 grid with 280,675 cells. The suitability data were divided into four grades: highly suitable, suitable, marginally suitable, and unsuitable. The land-use was reclassified into 9 types: arable land, orchard, forest, grass, urban, rural settlements, mining area, roads, water, and barren land.

**Table 2:** Data for the PSOLA model.

Data	Source
Land-use map, road map	Second National Land Survey (2007–2009)
DEM, slope map	ASTER GDEM V1 (2009, 30 m, http://giscloud.cn/)
Suitability data^a^, urban growth boundaries	Gaoqiao Town Land Use General Plan (2010–2020)
Socioeconomic data	Published statistical yearbooks (1996–2010, Bureau of Statistics)

**^a^**Suitability data include the suitability evaluation map of arable land, orchard, forest, and construction.

## 5. Results and Discussion

### 5.1 Model comparison

As described in Section 3, the PSOLA model has two improvements: the patch-level operations and the knowledge-informed rules. To verify the effectiveness of these improvements, we built four models with different improvement strategies as follows:

- Model A: with patch-level operations and knowledge-informed rules;
- Model B: with patch-level operations;
- Model C: with knowledge-informed rules;
- Model D: basic PSO.

The models were configured with the same parameters as shown in Table 3.

**Table 3:** Parameters of Models A, B C, and D.

*w*	*c*_*1*_	*c*_*2*_	*w*_*1*_	*w*_*2*_	*w*_*3*_	Population	Iteration
1.0	2.0	2.0	0.33	0.33	0.33	128	50

**Fig 6:**
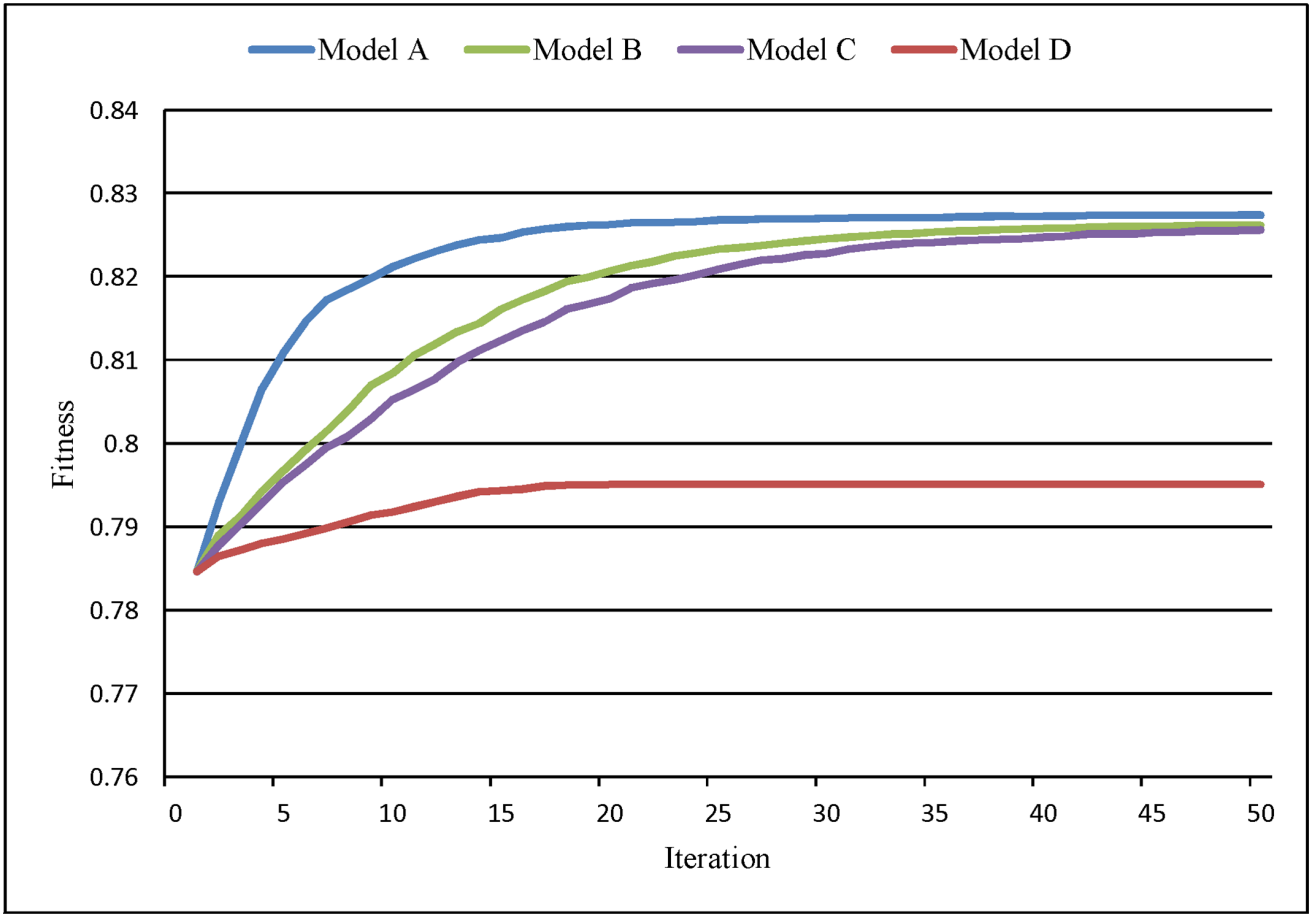
Convergence curves of Models A, B, C, D.

The convergence curves (Fig 6) show that Model A results in the highest fitness, while Model D has the lowest. Model D converges after 15 iterations, while Model A needs 20 iterations. Models B and C have the slowest convergence speeds, requiring 40 iterations. Model A balances the needs of efficiency and effectiveness. Models B and C both have a good fitness at the price of low convergence efficiency. Model D converges prematurely, resulting in its remarkable disparity of fitness compared to the others. Hence, through the comparative analysis of the convergence curves, it is evident that the improvements are effective.

For more evidence, we also examined the statistics of the result maps of the four models (Table 4). Model A dominates the performance in the terms of the social, economic, ecological, and overall benefits. The increases are 3.60%, 7.10%, 1.53% and 4.06%, respectively, when model A is compared with model D. The time consumption of model A is 24.01% more than the fastest model C, as the improvements increase the computation complexity. Model B performs slightly worse than model A but surpasses model C. This is because the metrics evaluating the social and ecological benefits promote compact development, and the patch-level operation in model B preserve the patchiness of the land-use landscape to avoid fragmentation and propel intensive land use. Model C attains 99.79% of the fitness of model A while only needing 80.64% of the time consumption. This indicates that the knowledge-informed rules help the model efficiently optimize land use. Model D, without any improvements, performs the worst in every aspect. However, it is odd that model D does not spend the least time, although it has the lowest computation complexity. The reason is that the algorithm will check if a land-use transition is suitable before it accepts it. Without the guidance of knowledge-informed rules, model D often fails to convert land use and dissipates much time on this checking procedure.

**Table 4:** Statistics for the result maps of Model A, B, C, D.

Model	*F*	*f*_*social*_	*f*_*economic*_	*f*_*ecological*_	Time(s)^a^
A (both improvements)	0.827408	0.776701	0.873476	0.857119	320.21
B (patch-level units)	0.826208	0.775629	0.873476	0.854555	305.68
C (knowledge-informed rules)	0.825637	0.774915	0.873476	0.853540	258.21
D (basic PSO)	0.795118	0.749695	0.815549	0.844205	273.11
Status quo	0.784666	0.743359	0.794207	0.840209	—

^a^All benchmark tests were performed on a Dell PowerEdge R910 (Xeon E7520*4/16GB).

From a comparative analysis of the models, it is clear that the improved PSOLA model shows a significantly enhanced ability to solve complicated land-use allocation problems, with better effectiveness.

The local government have provided a favorable quantity structure in Gaoqiao Land Use Planning (2006–2020). It says: the area of arable land should greater than 1871.50 ha, orchard should be less than 394.75 ha, forest should be greater than 6814 ha, and construction land (including urban and rural settlements) should be less than 996.94 ha. Table 5 shows that Model A performs better than Model B, C, and D in fulfilling the quantity constraints. So, it is another sign that our improvements in patch-level operations and knowledge-informed rules are effective.

**Table 5:** Quantity structures of the result maps of Model A, B, C, D (Unit: ha).

	Current	Model A	Model B	Model C	Model D	Planning
Arable land	1667.69	1863.31	1804.06	1797.06	1674.31	> = 1871.50
Orchard	681.25	415.63	477.38	488.00	639.88	< = 394.75
Forest	6654.00	6772.19	6748.00	6744.25	6698.13	> = 6814.00
Urban	162.81	179.06	179.06	179.00	169.13	< = 996.94*
Rural settlements	828.25	783.38	785.50	785.69	812.56	

*Including the area of urban and rural settlements

Percent Correct Match (PCM; [49, 50]) can be used to quantify the goodness-of-fit between a reference map and a simulated map. Although there is no reference map in our optimization model, but if we set the result map of Model A as the reference map, PCM will stand for the similarity between Model A and other models. If the PCM is large, it means that the result maps are identical in four models, leading to the conclusion that our improvements are unnecessary and invalid. On the other hand, if the PCM is low, it means that the result maps are different, proving that our improvements are indeed effective.

**Fig 7:**
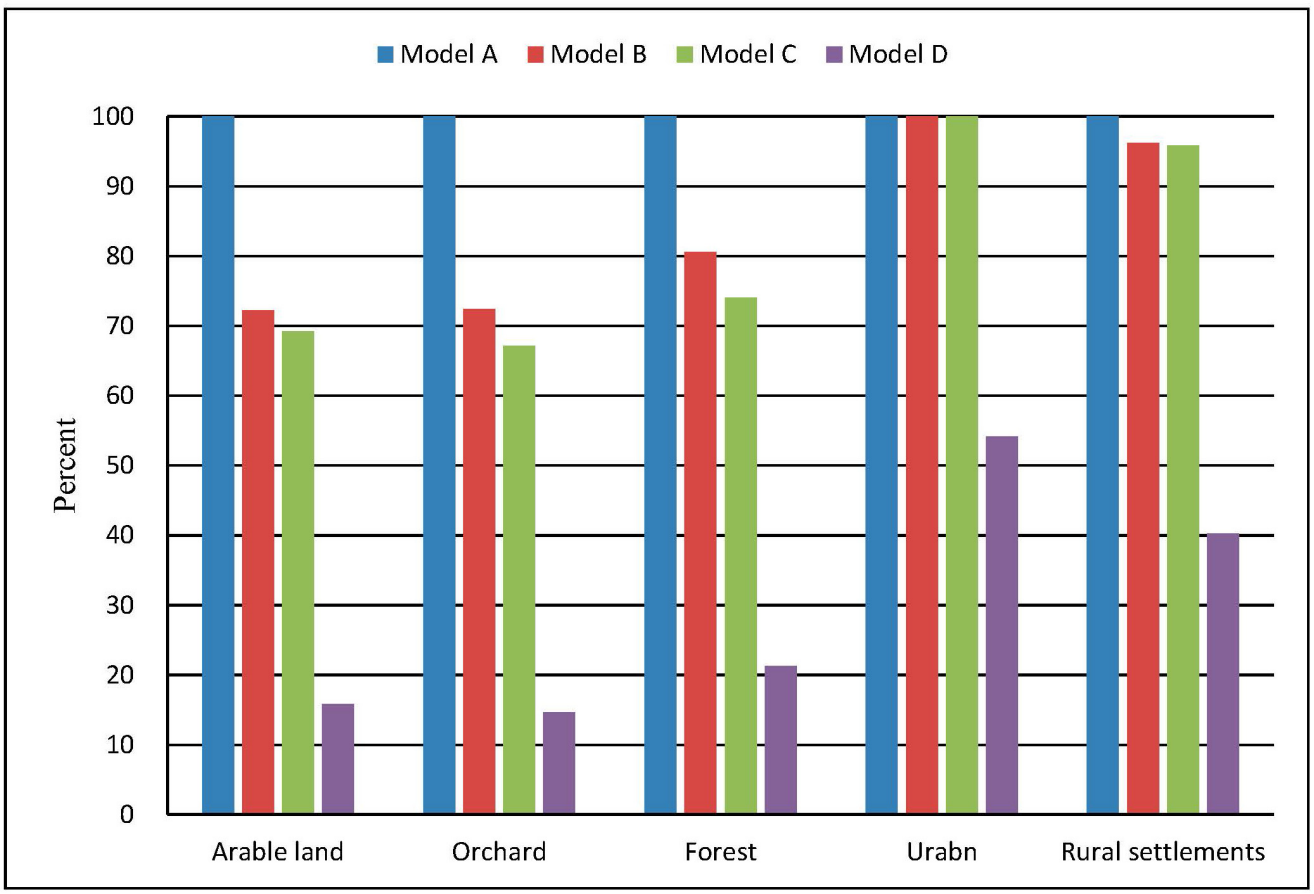
Histogram of PCM_Ps of land uses in Model A, B, C, D.

In Fig 7, it clearly shows that Model B, C, and D have lower PCM_Ps than Model A. Model B and C have lower PCM_Ps of arable land, orchard, and forest. Model D, which is a random model, performs the worst in all five land use types. As noted that, all the PCM_Ns are close to 100% for the reason of the most area keeping unchanged. All in all, the disparities of the PCM_Ps reveals that our improvements have taken effects on the result maps.

### 5.2 Model effectiveness analysis

**Fig 8:**
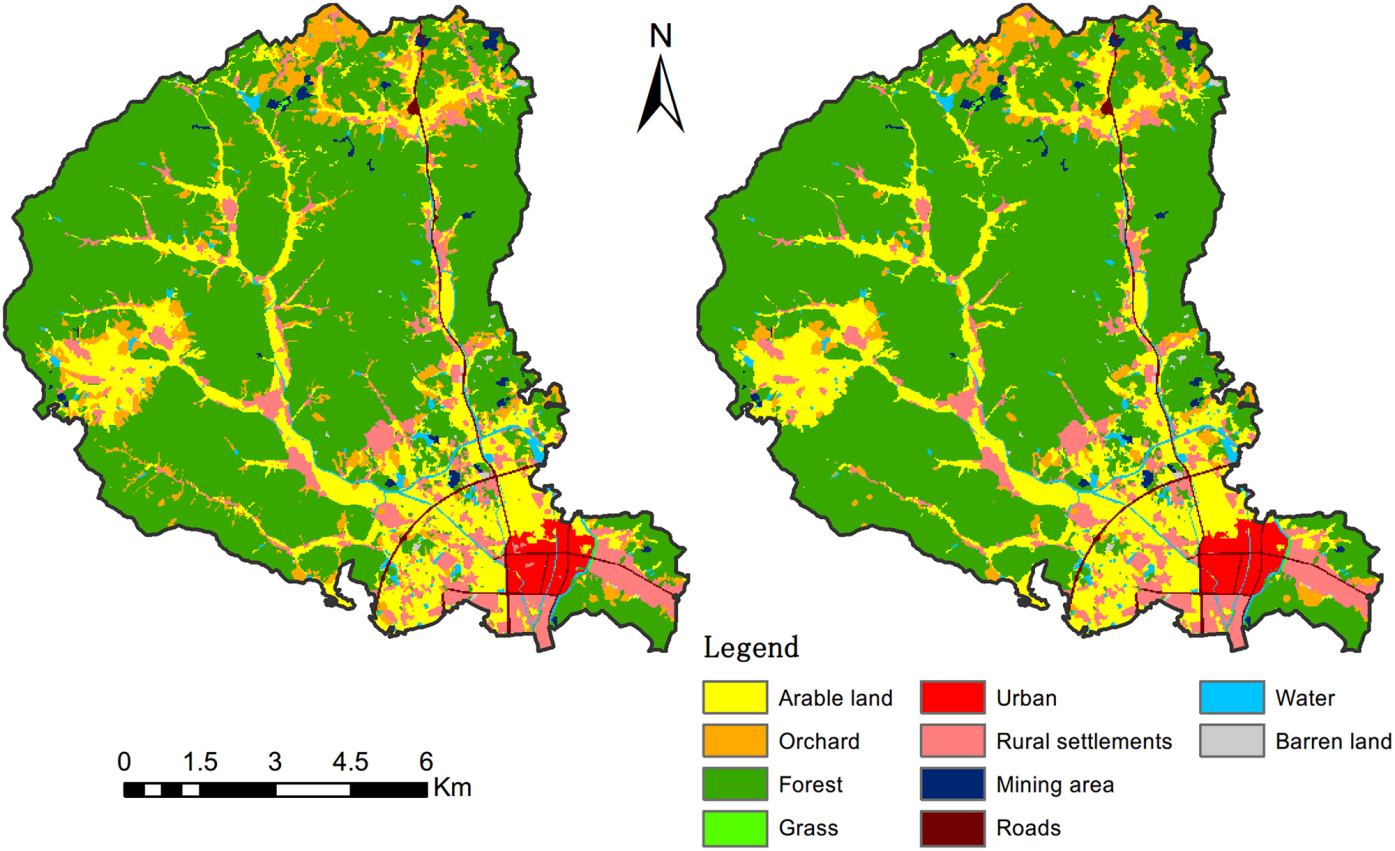
Land use status quo versus optimal solution.

A reasonable land-use allocation model should generate global optimized schemes while guaranteeing a rational land-use pattern in local areas. In this section, we will analyse the effectiveness of the model on both global and local scales through a comparative analysis of the land use status quo and the optimal solution (Fig 8).

**Fig 9:**
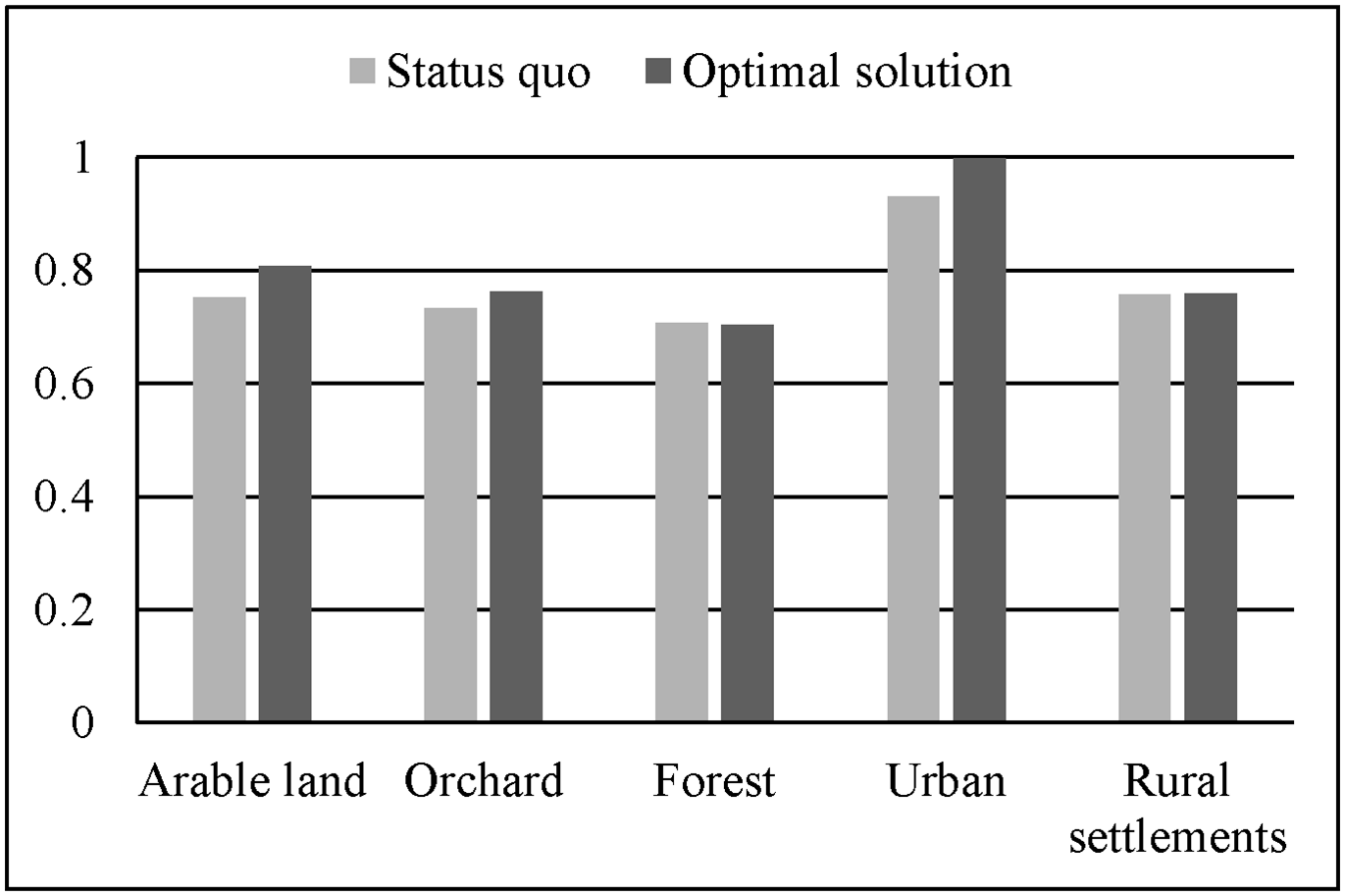
Suitability of the status quo and optimal solution.

**Fig 10:**
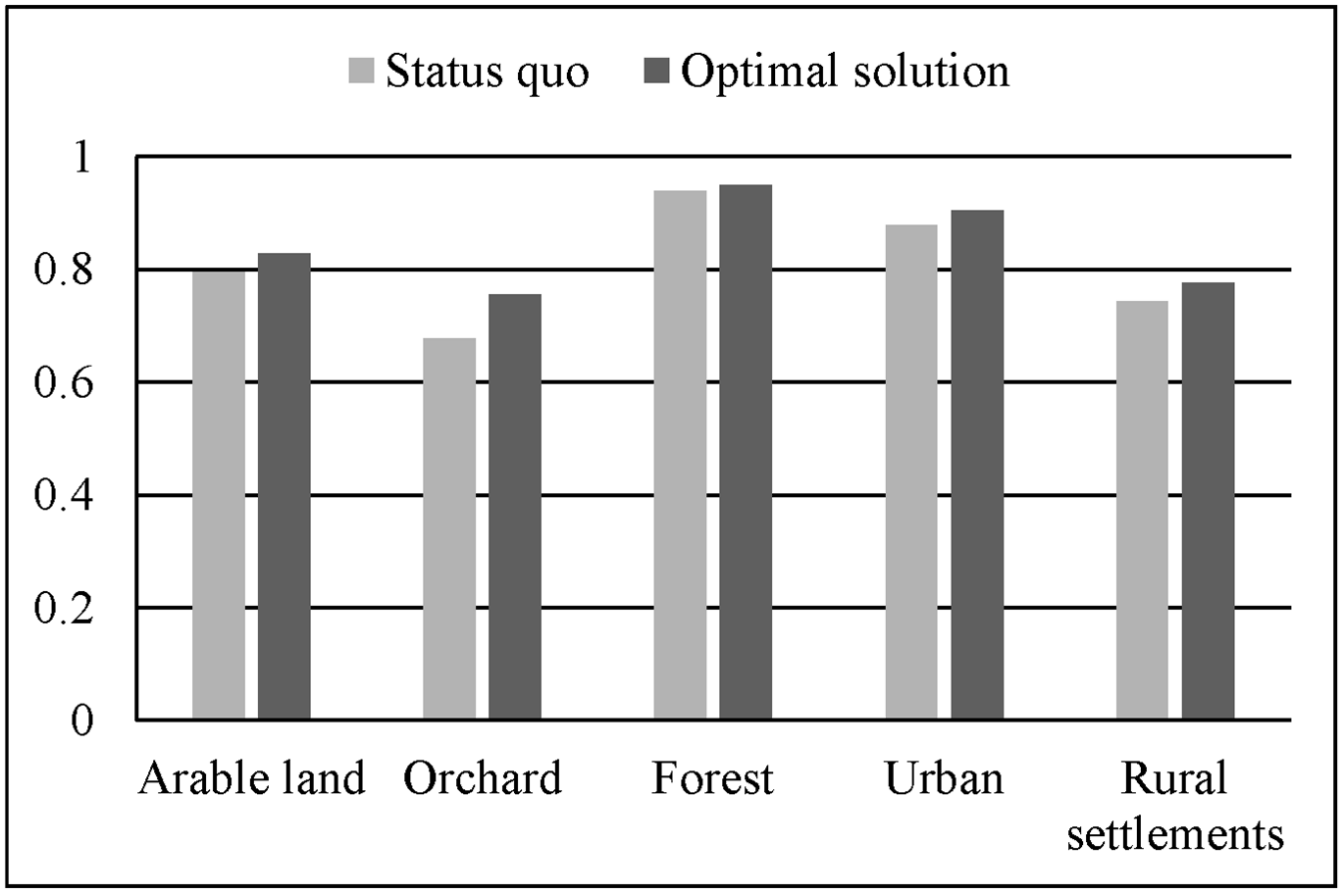
Compactness of the status quo and optimal solution.

In the global aspect, the optimal solution attains more benefits than the status quo. The increases are 4.49%, 9.98%, 2.01% and 5.45% for the social, economic, ecological and overall benefits, respectively (Table 4). The land use conversion matrix (Table 6) shows that the areas of arable land, forest and urban zones are increased by 11.73%, 1.48%, and 9.98%, while orchard and rural settlements decreased by 38.99% and 5.37%, respectively. The quantity of arable land is enlarged for food security for the future population growth. The urban area is expanded to support economic development. The rural settlements are decreased because of the consolidation of scattered villages. A large percentage of the orchard is converted to arable land and forest. This is because the local government has a priority to develop the fruit industry, which leads to an inferior status when orchards conflict with other land uses. We also evaluated the suitability and compactness of the status quo and optimal solution (Figs 9 and 10). The graphs indicate that the optimal solution is more suitable and compact than the status quo.

**Table 6:** Contingency table of land-use conversions.

Status quo	Optimal solution (ha)					
	Arable land	Orchard	Forest	Urban	Rural settlements	Total
Arable land	1625.88		26.31	10.94	4.56	1667.69
Orchard	177.81	378.50	123.56	0.81	0.56	681.25
Forest	11.81	37.13	6602.31	0.38	2.38	6654.00
Urban				162.81		162.81
Rural settlements	47.81			4.13	776.31	828.25
Total^a^	1863.31	415.63	6752.19	179.06	783.81	9994.00

^a^Undeveloped land-uses such as grass, mining area, roads, water, and barren land are excluded.

**Fig 11:**
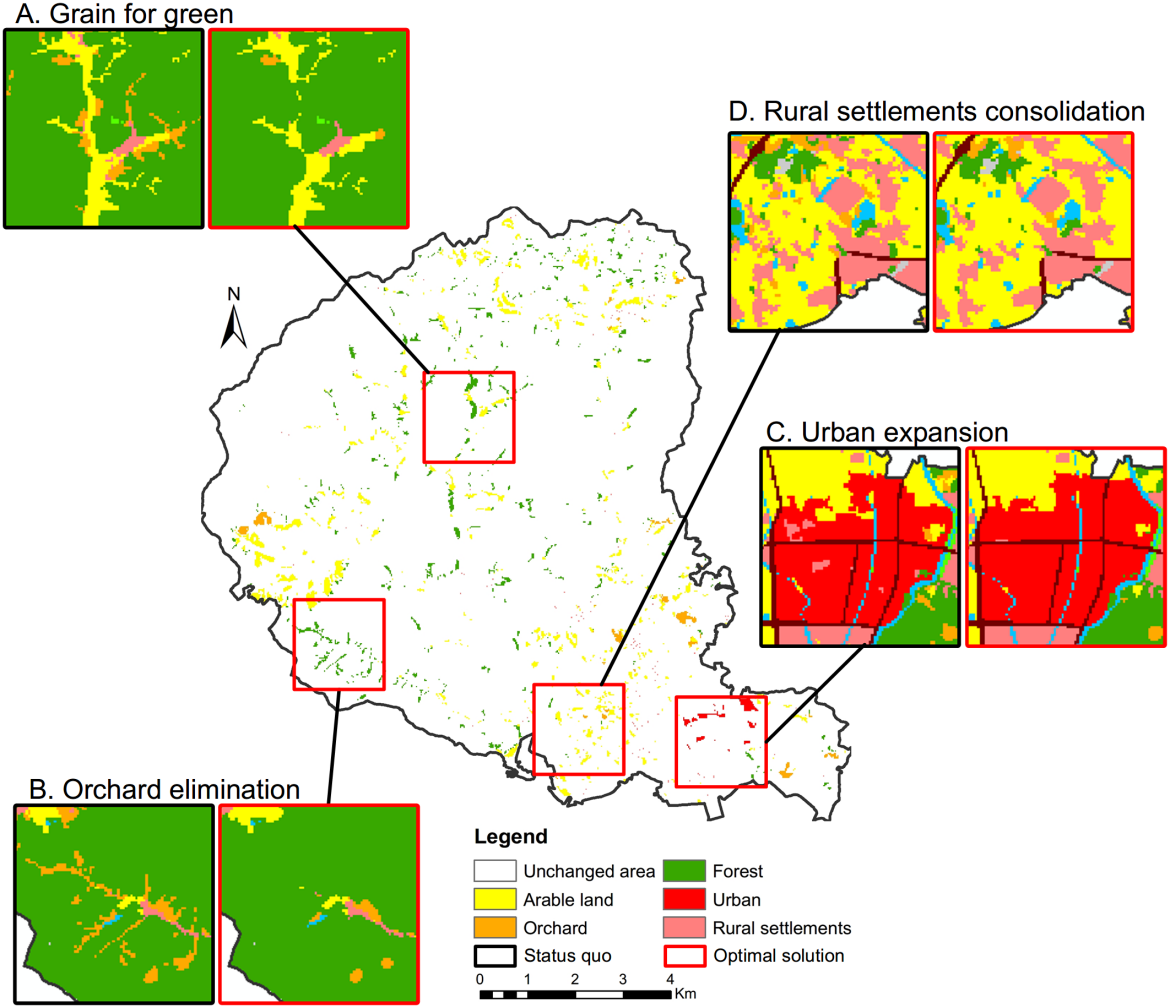
Areas of change between the status quo and optimal solution.

In the local aspect, we made a cell-by-cell comparison to determine the disparity between the status quo and optimal solution (Fig 11). The conversion rates are 2.51%, 44.44%, 0.78%, 0.00% and 6.27% on arable land, orchard, forest, urban, and rural settlements, respectively. Due to the Grain for Green policy, a 26.31 hectares of arable land whose slope is greater than 25 degrees was converted to forest (Fig 11A). A large amount of orchard with poor road accessibility was transformed to arable land and forest (Fig 11B). The urban area expanded to the north under the constraint of the urban growth boundaries (Fig 11C). Some small scattered villages are merged into the nearest large rural settlements to reduce land consumption (Fig 11D). Through the analysis of the changed areas, it is confirmed that the local land-use transitions are rational and reasonable by following the predefined knowledge-informed rules (Table 1).

According to the two aspects of the analysis, the optimal solution shows overall benefits on the global scale and rational land-use patterns in the local areas. Therefore, we can conclude that the PSOLA model is effective for both global and local-scale optimization.

## 6. Conclusions

In this study, we developed a new land-use allocation model with PSO. In the new PSOLA model, two significant improvements have been made to overcome its shortcomings. One improvement is applying patch-level operations. Compared to the conventional cell-level operations, patch-level operations preserve landscape patchiness to avoid fragmented and extensive land-use pattern. The other is introducing knowledge-informed rules to provide land-use planning knowledge to guide land-use transitions. The knowledge-informed rules are extracted from a questionnaire survey administered to departments of land management and expert in relevant fields.

To validate the PSOLA model, Gaoqiao Town in China was used as the study area. Four models with different improved strategies were implemented with same parameters. The results show that the model with patch-level operations and knowledge-informed rules could generate a better solution than the others. Therefore, it is concluded that these two improvements enhance the ability to solve land-use allocation problems, with a better efficiency and effectiveness. To further evaluate the effectiveness of the model, a comparative analysis of the status quo and optimal solution was made on the aspects of global optimization and local rationality. The optimal solution shows better overall benefits on the global scale and rationality in local areas. Overall, this case study confirms the capacity of the model to handle complex land-use allocation problems. The new PSOLA model could provide a useful tool to support decision making in land-use planning.

Although the model has shown its practicability in land-use planning, it still has some shortcomings. The objectives in the model are inadequate to describe the goal of regional development. The knowledge-informed rules are also not comprehensive to meet all requirements for decision makers. Due to the diversity of regional development preferences, it is impossible to enumerate all of the knowledge-informed rules. However, the model has an open and extensible architecture. It is convenient for modelers to define appropriate objectives and knowledge-informed rules according to actual conditions. Hence, we are glad to see that the PSOLA model (http://jingsam.github.io/PSOLA/) could help planners and researchers solve their land-use allocation problems.

## Supporting Information

S1 Code Source code of the PSOLA model. The source code is also available on http://jingsam.github.io/PSOLA/.
(ZIP)

S1 Dataset Dataset used in the case study. This dataset includes the land-use map (DLTB.tif), digital evaluation model (DEM.tif), slope (SLOPE.tif), road accessibility (ROAD.tif), urban boundaries (URBAN.tif), suitability of arable land (GDSYX.tif), suitability of orchard (YDSYX.tif), suitability of forest (LDSYX.tif), suitability of construction (JSSYX.tif).
(ZIP)
