# Supplementary figures and images for: PSOLA: A Heuristic Land-Use Allocation Model Using Patch-Level Operations and Knowledge-Informed Rules
Source: PLoS One. 2016 Jun 20;11(6):e0157728. doi: 10.1371/journal.pone.0157728 (PMC4913917; doi:10.1371/journal.pone.0157728)

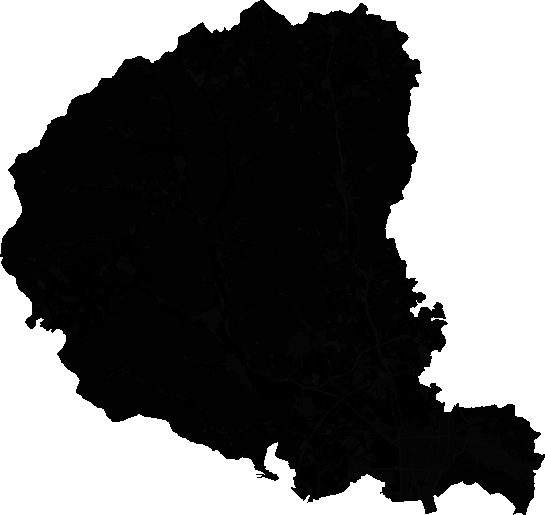

Supplement: S1 Code — The source code is also available on http://jingsam.github.io/PSOLA/. (ZIP) [file pone.0157728.s001.zip › PSOLA-master/example/gq/data/DLTB.tif]

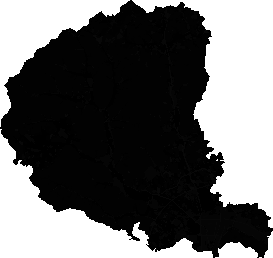

Supplement: S1 Code — The source code is also available on http://jingsam.github.io/PSOLA/. (ZIP) [file pone.0157728.s001.zip › PSOLA-master/example/gq/data/DLTB.tif.ovr]

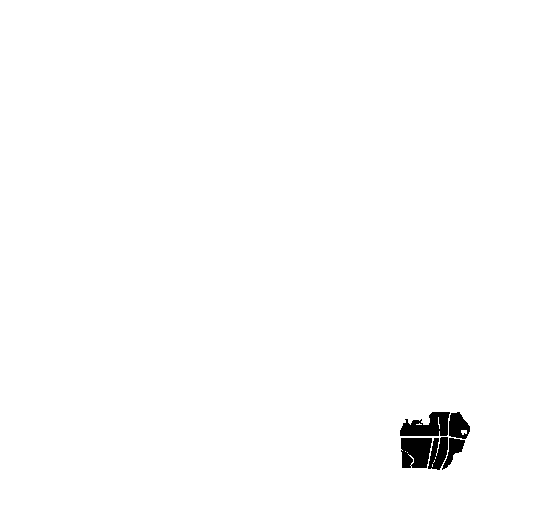

Supplement: S1 Code — The source code is also available on http://jingsam.github.io/PSOLA/. (ZIP) [file pone.0157728.s001.zip › PSOLA-master/example/gq/data/URBAN.tif]

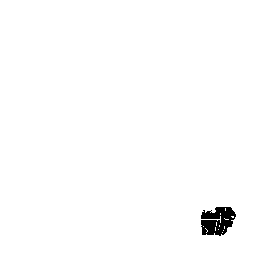

Supplement: S1 Code — The source code is also available on http://jingsam.github.io/PSOLA/. (ZIP) [file pone.0157728.s001.zip › PSOLA-master/example/gq/data/URBAN.tif.ovr]
